# The treatment of glioblastoma multiforme through activation of microglia and TRAIL induced by rAAV2-mediated IL-12 in a syngeneic rat model

**DOI:** 10.1186/1423-0127-19-45

**Published:** 2012-04-22

**Authors:** Tsung-Lang Chiu, Mei-Jan Wang, Chin-Cheng Su

**Affiliations:** 1 Tzu-Chi University, Hualien, Taiwan; 2 Division of Neuro-Oncology, Neuro-Medical Scientific Center, Buddhist Tzu-Chi General Hospital, Hualien, Taiwan; 3 Department of Research, Buddhist Tzu Chi General Hospital, Hualien, Taiwan; 4 Tzu Chi College of Technology, Hualien, Taiwan, ROC; 5 Department of surgery and Comprehensive Breast cancer center, Changhua Christian Hospital, Changhua, Taiwan

## Abstract

**Background:**

Microglial cells are the predominant immune cells in malignant brain tumors, but tumors may release some factors to reduce their defensive functions. Restoration of the anti-cancer function of microglia has been proposed as a treatment modality for glioblastoma. We examined the effect of intra-cranially administered recombinant adeno-associated virus encoding interleukin-12 (rAAV2/IL12) on transfection efficiency, local immune activity and survival in a rat model of glioblastoma multiforme.

**Methods:**

F344 rats were injected with rAAV2/IL12 and implanted with syngeneic RG2 cells (glioblastoma cell line). Intracerebral interleukin-12 and interferon-γ concentrations were determined by ELISA. Activation of microglia was determined by expressions of ED1 and tumor necrosis factor-related apoptosis-inducing ligand (TRAIL) which were evaluated by Western blotting and immunohistochemistry. The proliferation of cancer cells was evaluated with Ki67 immunohistochemistry and apoptosis of cancer cells with TUNEL.

**Results:**

The brains treated with rAAV2/IL-12 maintained high expression of interleukin-12 and interferon-γ for at least two months. In syngeneic tumor model, brains treated with rAAV2/IL12 exhibited more infiltration of activated microglia cells as examined by ED1 and TRAIL stains in the tumor. In addition, the volume of tumor was markedly smaller in AAV2/IL12-treated group and the survival time was significantly longer in this group too.

**Conclusion:**

The intra-cerebrally administered rAAV2/IL-12 efficiently induces long lasting expression of IL-12, the greater infiltration of activated microglia cells in the tumor associated improved immune reactions, resulting in the inhibited growth of implanted glioblastoma and the increased survival time of these rats.

## Background

Interleukin-12 (IL-12) is a potent anti-cancer cytokine that enhances innate and adaptive immune-responses. At the molecular and cellular level, IL-12 facilitates maturation of natural killer cells and cytotoxic T cells, stimulates secretion of interferon-γ (IFN-γ) leading to anti-angiogenesis, enhances secretion of the tumor necrosis factor (TNF) superfamily leading to apoptosis, and provokes antigen-specific adaptive immunity of tumors via the T_H_1 pathway [1-4]. However, systemic administration of recombinant IL-12 protein is associated with a high risk of injury to vital organs [5,6]. Several preclinical studies, therefore, have attempted to transfer IL-12 by direct injection into tumors or tumor environments to reduce systemic toxicity [7,8].

Clinical trials have shown that gene transfer via an adeno-associated virus (AAV) can be safe and effective. In 1996, AAV-mediated gene transfer of the normal cystic fibrosis transmembrane conductance regulator gene was used to treat humans with cystic fibrosis [9], and subsequent studies improve the therapeutic efficacy of this treatment. In recent years, rAAV type 2 (rAAV2) has been used to transfer the glutamic acid decarboxylase gene into the subthalamic nucleus of patients with advanced Parkinson's disease [10]. In 2010, rAAV2-mediated transfer of the normal RPE65 gene into the retina of patients with Leber's congenital amaurosis promoted expression of the transferred gene for at least one year and patients experienced satisfactory therapeutic responses [11].

Microglia cells play an important role in the development of neoplasms in the central nervous system (CNS). In healthy patients, microglial cells comprise 2-20% of CNS cells and eliminate invading microbes or transformed cells via major histocompatibility complex type 2 and/or phagocytosis [12,13]. However, in the presence of a tumor, microglial cells infiltrate the tumor and can constitute 10-34% of tumor cells and contribute to tumor growth [14,15]. Tumors can secrete transforming growth factor-β, matrix metaloproteases, and chemokines that regulate the function of tumor-associated microglial cells, and allow the tumor to continue to grow, develop, and metastasize [16-18].

In this study, we developed a strategy for treatment of gliobastoma multiforme in a rat model that employs intracranial injection of rAAV2 that encodes IL-12. Our goal was to extend the therapeutic window and reduce systemic toxicity. The anti-cancer effects were evaluated by measuring generation of IL-12, IFN-γ, and tumor necrosis factor-related apoptosis-inducing ligand (TRAIL), and induction of activated microglia cells.

## Methods

### Construction of rAAV2 encoding IL-12

The full length cDNA of rat IL-12 (InvivoGen, Sandiago, USA) or enhanced green fluorescent protein (GFP, InvivoGen, Sandiago, USA) was amplified using the polymerase chain reaction (PCR) and subcloned into pAAV-MCS (Stratagene, La Jolla, California, USA). The sequence was verified by DNA sequencing (Protec, Oberstenfeld, Germany). The procedures for creation of rAAV2 encoding IL-12 (rAAV2/IL12) or GFP (rAAV2/GFP) include 5 main steps: transformation of *E. coli*, plasmid extraction, culturing of HEK 293 cells, packaging of rAAV2/IL12 or rAAV2/GFP in HEK 293 cells, and rAAV2/IL12 or rAAV2/GFP purification [19]. In brief, *E. coli *(ECOS 101) cells were used as competent cells, and were transformed by pAAV-RC (Stratagene), pHelper (Stratagene), and pAAV-IL-12 or rAAV2/GFP, then cultured at 37°C in 2YT-Broth A50 (Invitrogen, Carlsbad, USA) with ampicillin (50 μg/mL). Transformed *E. coli *cells were resuspended in 2YT-Broth A50 and prepared for extraction of the three plasmids. Plasmid DNAs were purified with a plasmid Mega preparation kit (Qiagen, Duesseldorf, Germany), and the extracted plasmids were quantified by measuring the 260 nm/280 nm absorbance ratio. Frozen HEK 293 cells were thawed at 37°C and cultured with DMEM (Cellgro, Manassas, USA), 10% FBS (Hyclone, Logan, USA), and 1% penicillin/streptomycin (Gibco, Carlsbad, USA). The extracted plasmids, pAAV-RC, pAAV-IL-12 (or pAAV2-GFP), and double the amount of pHelper, were mixed and shaken with CaCl_2_. The mixture was spread onto HEK 293 cell culture dishes and incubated with DMEM, 10% FBS, and 1% P/S at 37°C for rAAV/IL12 or rAAV2/GFP packaging. Finally, the packaged rAAVs were purified on a heparin column (Heparin Actigel Sterogen, San Gabriel, USA).

### Animal model

The animal use protocol was reviewed and approved by the Institutional Animal Care and Use Committee of Tzu-Chi Hospital. F344 rats were purchased from the NLAC (National Laboratory Animal Center, Taipei, Taiwan) and bred in the Laboratory Animal Center of Tzu-Chi University for acclimatization at least 7 days before initiation of experiments. The F344 rat syngeneic GBM cell line RG2 was purchased from FIRDI (Food Industry Research and Development Institute, Hsinchu, Taiwan).

### Injection of rAAV2/IL12 and implantation of RG2 cells

F344 rats (male, bodyweight, 200-250 g) were anesthetized by intra-abdominal injection of diluted chloral hydrate (40 mg/mL) at a dose of 1 mL/100 g bodyweight. The head of the rat in prone position was fixed in the stereotactic frame (Lab Standard™ Stereotaxic Instrument, Wood Dale, USA). Craniostomy was performed under the guidance of stereotactic frame with a target of 3 mm right and 3 mm behind the bregma. A total of 30 μL rAAV2/IL12 or rAAV2/GFP (1.96 × 10^12 ^particles/mL) was injected 4 mm deep into the brain via a 50 μL syringe at a rate of 5 μL/min. Ten minutes after completion of the injection, the syringe was slowly withdrawn, the craniostomy was sealed with bone wax, and the wound was closed with 3-0 nylon. Two weeks later, 500 RG2 cells in 10 μL solution were injected into rat brain, under stereotactic guidance, from the same burr hole, but 1 mm anterior to virus injection site.

### ELISA of IL-12 and IFN-γ

After brains were harvested, 3 mm-thick slices were cut using a rat brain-cutting apparatus. Total proteins were separately extracted from brain sections of the right and left hemispheres composed of maximal tumor volume (Figure 2E). The concentrations of IL-12 and IFN-γ were determined with ELISA kits (R&D Systems, Minneapolis, USA) according to the manufacturer's instructions. Briefly, the extracted lysates from brain sections were incubated in 96-well microplates coated with anti-rat primary antibodies, then developed with secondary antibodies conjugated with horseradish peroxidase. After adding the substrate and stop solution, an Emax Precision Microplate Reader (Molecular Devices, Silicon Valley, USA) was used to measure absorbance at 450 nm.

**Figure 1:**
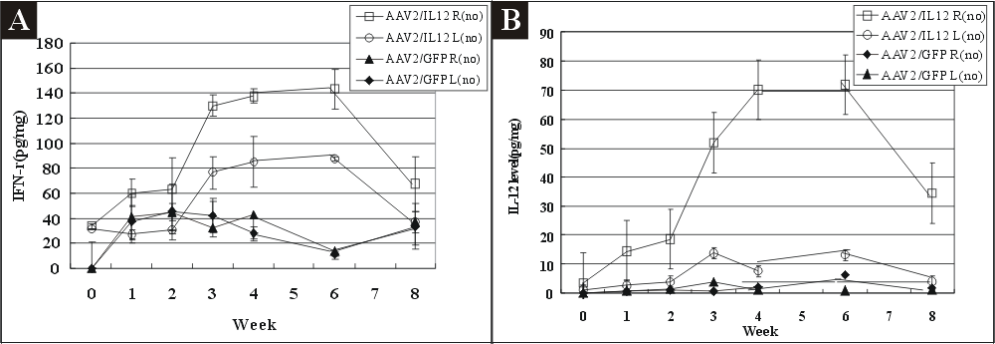
**Concentrations of IL-12 and IFN-γ in right (R) and left (L) hemispheres of mice treated with rAAV2/GFP (n = 14) or rAAV2/IL-12 (n = 14)**. All mice were not implanted with tumor as indicated by (no). We measured both IFN-γ and IL-12 at the same time. IFN-γ (A) and IL-12 (B) levels, as expressed in picogram in 1 mg extracted protein, were measured by ELISA on the last day of week 0, 1, 2, 3, 4, 6, 8 (n = 2 each week) after injection of the virus vectors. All virus vectors were injected on the right hemisphere for this and the following experiments. For the following experiments in tumor implantation, 500 cells of RG2 (GBM cell line) were injected into the brain 1 mm anterior to the vector injection site on the last day of week 2 after the viral injection.

**Figure 2:**
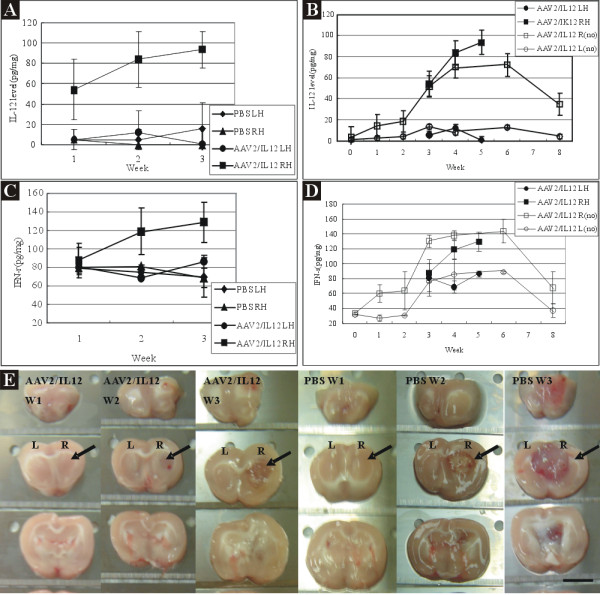
**Concentrations of IL-12 and IFN-γ in right (R) and left (L) hemispheres of rats treated with PBS or rAAV2/IL-12 and with tumor implantation**. Concentrations of IL-12 (A) and IFN-γ (C) were measured on the last day of week 1, 2, and 3 post tumor implantation (i.e., on the last day of week 3, 4, and 5 post viral injection). The concentrations of IL-12 (A) and IFN-γ (C) with tumor transplantation were compared to those of IL-12 (B) and IFN-γ (D) with no tumor implantation (no). Note that both concentrations with no tumor implantation were taken from the data in Figure 1. Photographs of 2 mm thickness brain sections *(E) *show rAAV2/IL12-treated (left 3 columns) and PBS-treated (right 3 columns) brains harvested on the last day of week 1 (W1), week 2 (W2), and week 3 (W3) post tumor implantation. Black arrows indicate the maximal tumor size and this site was taken for analyzing the concentration of IL-12 and IFN-γ. PBS LH (diamond): left hemisphere from the PBS-treated; PBS RH (triangle): right hemisphere from the PBS-treated; AAV2/IL12 RH (round): right hemisphere from the rAAV2/IL12-treated; AAV2/IL12 LH (square): left hemisphere from the rAAV2/IL12-treated; AAV2/IL12 R(no) (empty square): right hemisphere of rAAV2/IL12-treated brain without tumor implantation; AAV2/IL12 L(no) (empty round): left hemisphere from the rAAV2/IL12-treated brain without tumor implantation. The scale bar indicates 4 mm.

### Western blotting of ED1 and TRAIL

A total of 20 μg protein was extracted from the brain slices of the right and left hemispheres for Western blotting. The activated microglia marker ED1 and TRAIL were checked. After the procedure of SDS-PAGE, proteins were blotted onto a PVDF membrane and then the membrane was probed with monoclonal mouse anti-rat ED1 (mouse monoclonal antibody to CD68, AbD Serotec, Kidlington, UK) or polyclonal goat anti-rat TRAIL antibody (Santa Cruz Biotechnology, USA) at a ratio of 1:100. The membrane was further incubated with a secondary peroxidase-labeled antibody and quantified by chemoluminescence.

### Immunohistochemistry of TRAIL, activated microglia cells, Ki67, and TUNEL

Brains were fixed in 4% formaldehyde and dehydrated. The brain was cut into 3 mm thick slices with a rat brain-cutting apparatus. The brain slices with maximal tumor growth were further cut into 12 μm thick sections. These brain sections, after rehydration and permeabilization with a buffer solution, were then placed on slides and prepared for staining.

For immumohistochemistry, these brain sections were immersed in 3% hydrogen peroxide (to quench activity of endogenous peroxidase) and then incubated with monoclonal mouse anti-rat ED1 (AbD Serotec), polyclonal goat anti-rat TRAIL, Ki67 and TUNEL antibodies (Santa Cruz Biotechnology). Antibodies were quantified with the immunoperoxidase secondary detection system, according to the manufacturer's guidelines (Chemicon, Temecula, USA).

### Detection of nitric oxide (NO) after IL-12 stimulation in vitro

2 × 10^5 ^BV2 cells (mouse microglia) were implanted and cultured in each well of a 24 well plate. The cells were treated with 5 different conditions which included blank (negative control), 5 ng/ml IL-12, 10 ng/ml IL-12, 50 ng/ml IL-12, and LPS (positive control). After 24 hour incubation, 100 μl mixture of 1% Na solution and 1% Su solution were added in. After 10 minutes incubation, NO secretion was checked by detection of OD 500 (Molecular Devices, precision microplate reader).

### Experimental protocol

A total of 106 rats were used in this study (Table 1). They were divided into 6 groups.

**Table 1:** Grouping of experiment

	ELISA		ELISA		Western Blot		Immunohistochemistry			Tumor growth		Survival	
	**AAV2** **/IL12** **(no)#**	**AAV2** **/GFP** **(no)#**	**AAV2** **/IL12**	**PBS**	**AAV2** **/IL12**	**PBS**	**AAV2** **/IL12**	**AAV2** **/GFP**	**PBS**	**AAV2** **/IL12**	**PBS**	**AAV2** **/IL12**	**PBS**

Week 0	2	2											

Week 1	2	2	3	3	1	1				3	3		

Week 2	2	2	3	3	1	1				3	3		

Week 3	2	2	3	3	3	3	4	2	2	3	3		

Week 4	2	2											

Week 5													

Week 6	2	2											

Week 7													

Week 8	2	2											

Death												12	12

# (no) indicates no tumor implantation

Group 1 (n = 28):14 treated with AAV2/IL12 and 14 treated with AAV2/GFP. All rats were not implanted with tumor. The level of IFN-γ and IL-12 were detected by ELISA on the last day of week 0, 1, 2, 3, 4, 6, and 8 (n = 2 each week) post treatment.

Group 2 (n = 18):9 treated with AAV2/IL12 and 9 treated with PBS. Following these treatments, all rats were implanted with tumor (GBM). The level of IFN-γ and IL-12 were detected with ELISA on the last day of week 1, 2, and 3 (n = 3 each week) post tumor implantation (i.e., on the last day of week 3, 4, and 5 post viral or PBS).

**Figure 3:**
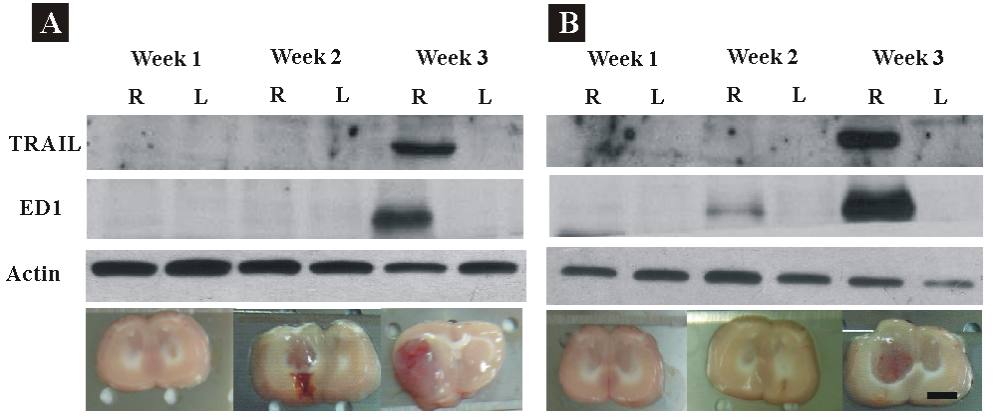
**Western blotting of ED1 (Marker of activated microglia) and TRAIL (tumor necrosis factor-related apoptosis-inducing ligand)**. Brains were treated with PBS or rAAV2/IL12 and then implanted with tumor on the last day of Week 2 post viral vector injection. The brains were used for Western blotting analysis of the expression of ED1, 34 kDa, and TRAIL, 104 kDa, from microglial cells on the last day of week 1, 2, and 3 following tumor implantation. The brain slices for Western blotting analysis are shown at the bottom. All brains were harvested on the last day of week 1, week 2, and week 3 post tumor implantation. The scale bars indicate 2 mm in brain sections.

Group 3 (n = 10):5 treated with AAV2/IL12 and 5 treated with PBS. Following these treatments, the rats were further implanted with tumor. Western blot was performed for TRAIL and ED1 on the last day of week 1 (n = 1, each group), 2 (n = 1, each group), and 3 (n = 3, each group) post tumor implantation. (Figure 3)

**Figure 4:**
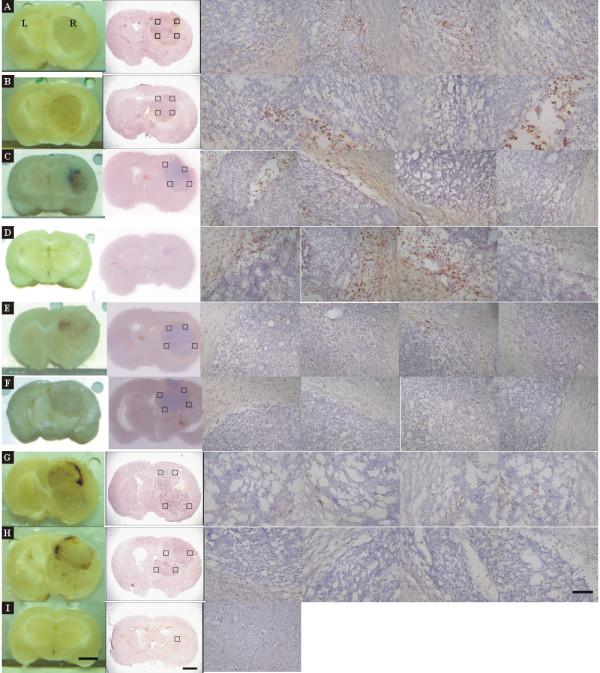
**Immunohistochemistry of ED1 stain in brain sections treated with rAAV2/IL12 (*A, B, C, D*, n = 4), AAV2/GFP (*E, F*, n = 2), or PBS (*G, H*, n = 2), accompanied with tumor implantation; and that in the brain section treated with nothing (n = 1)**. Immunohistochemistry of brain sections was performed for ED1 on the last day of week 3 after tumor implantation. The brain sections were stained with hematoxylin for nuclei and ED1 for activated microglial cells. The 1^st ^column shows the brain sections pictured before immunostaining; the 2^nd ^column shows the brain sections pictured after staining; the 3^rd ^to 6^th ^columns show pictures taken at four quadrants (black squares in 2^nd ^column) of the tumor adjacent to the normal tissue in the right hemisphere. ED1-positive cells show dark brown. The scale bars indicate 2 mm in 1^st ^and 2^nd ^columns and 100 μm in 3^rd^-6^th ^columns.

**Figure 5:**
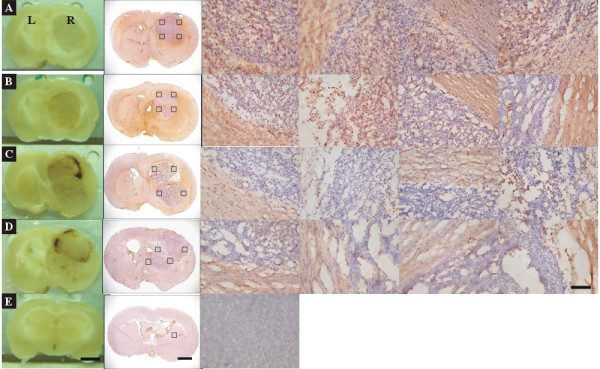
**Immunohistochemistry of TRAIL stain in brains treated with AAV2/IL12 (*A, B*) or PBS (*C, D*), accompanied with tumor implantation; and that in the brain treated with nothing (*E*)**. Brain sections used in Figure 4 were also stained with hematoxylin for nuclei and TRAIL, and pictured as in Figure 4. TRAIL-positive cells show dark brown. The scale bars indicate 2 mm in 1^st ^and 2^nd ^columns and 100 μm in 3^rd ^and 6^th ^columns.

**Figure 6:**
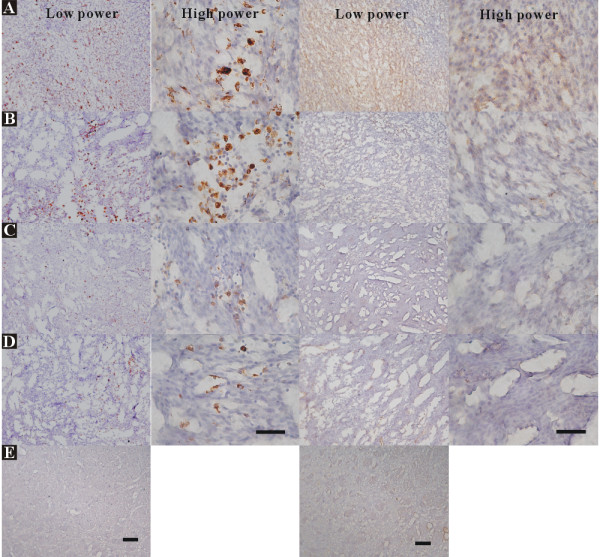
**Immunohistochemical stains of ED1 and TRAIL in brain sections treated with AAV2/IL12 (*A, B*) or PBS (*C, D*), accompanied with tumor implantation; and that in the brain treated with nothing (*E*)**. Brain sections used in Figure 4 were also stained with hematoxylin for nuclei, ED1, and TRAIL, and pictured as in Figure 4. The 1^st ^and 2^nd ^columns show the low and high power fields of ED1 stain, respectively; the 3^rd ^and 4^th ^columns show the low and high power fields of TRAIL stain. Cells stained with ED1 or TRAIL show dark brown. The scale bars indicate 100 μm in low power field and 50 μm in high power field.

**Figure 7:**
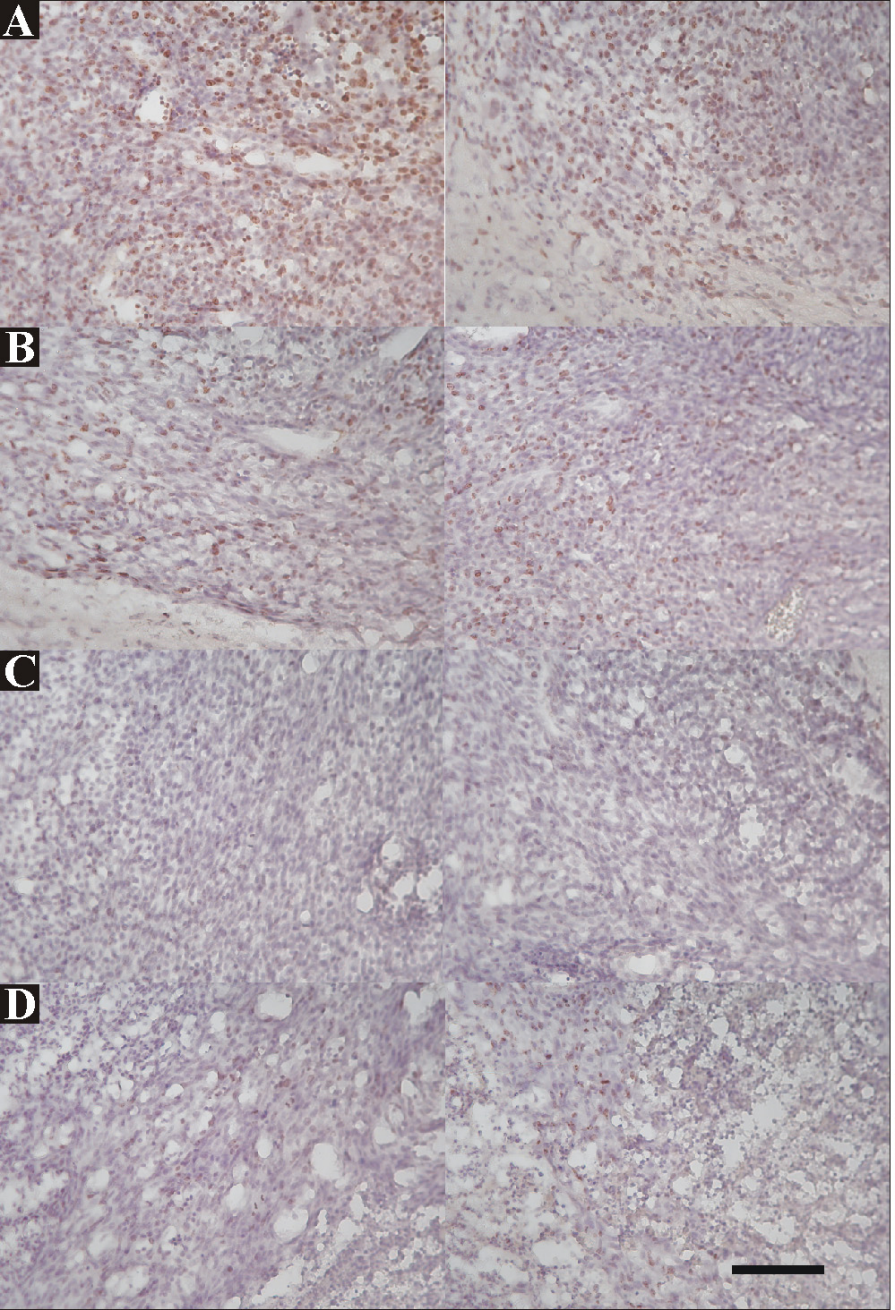
**Immunohistochemistry of Ki67, a cell proliferation marker, in brains treated with rAAV2/GFP (*A, B*) or rAAV2/IL12 (*C, D*), accompanied with tumor implantation**. On the last day of week-3 post tumor implantation, brain sections were harvested from two rAAV2/GFP-treated rats (*A, B*) and two rAAV2/IL12-treated rats (*C, D*). Cells stained with Ki67 show dark brown. The scale bar indicates 100 μm (low power field).

**Figure 8:**
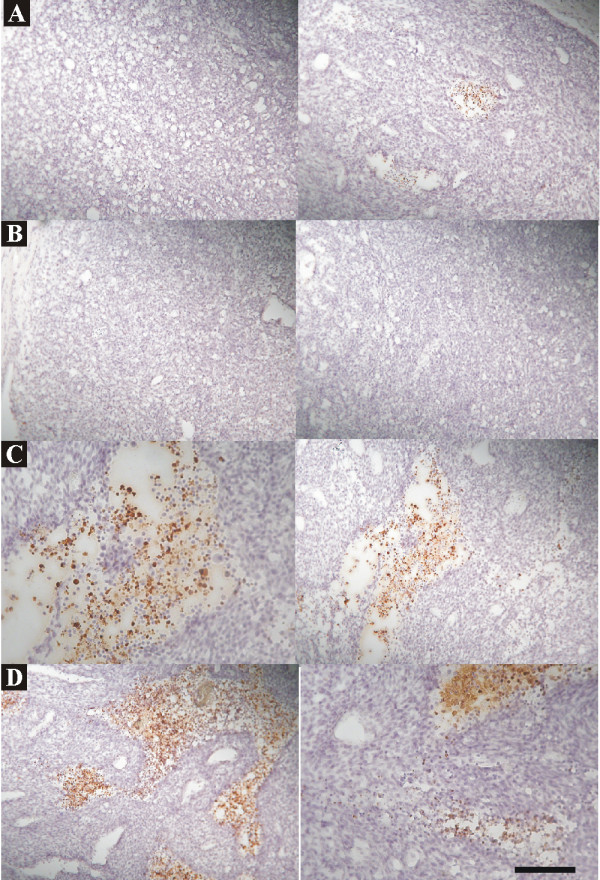
**Immunohistochemistry of TUNEL, an apoptotic cell marker, in brains treated with rAAV2/GFP (*A, B*) or rAAV2/IL12 (*C, D*), accompanied with tumor implantation**. On the last day of week-3 post tumor implantation, brain sections were harvested from two rAAV2/GFP-treated rats (*A, B*) and two rAAV2/IL12-treated rats (*C, D*). Apoptotic cells stained with TUNEL show dark brown. The scale bar indicates 100 μm (low power field).

Group 4 (n = 8):4 treated with AAV2/IL12, 2 treated with AAV2/GFP, and 2 treated with PBS. The treated rats were further implanted with tumor. Immunohistochemistry of brain sections of these rats was performed for cell markers including TRAIL, ED1, Ki67 and TUNEL on the last day of week 3 after tumor implantation. (Figures 4, 5, 6, 7 and 8)

**Figure 9:**
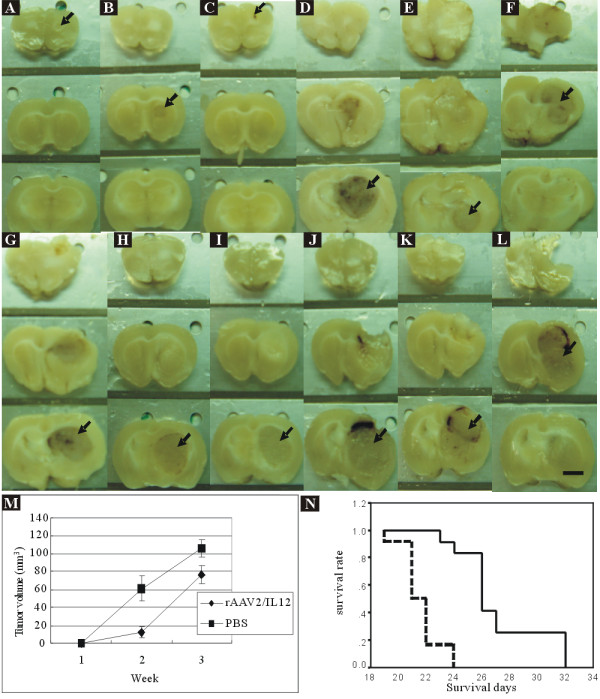
**Tumor growth and survival rate of rAAV2/IL12-treated and PBS-treated rats**. Tumor growth was evaluated with brain slices harvested on the last day of week-1 (n = 3 each group), week-2 (n = 3 each group), and week-3 (n = 3 each group) post tumor implantation. The black arrows indicate the brain sections composed of maximal tumor mass, for delineation and estimation of tumor volume. The scale bar indicates 2 mm. *A-C*. rAAV2/IL12-treated tumors grew for 2 weeks. *D-F*. PBS-treated tumors grew for 2 weeks. *G-I*. rAAV/IL12-treated tumors grew for 3 weeks. *J-L*. PBS-treated tumors grew for 3 weeks. *M*. Mean tumor volume was estimated with Axiovision Rel. 4.7, Carl Zeiss Microimaging, Stuttgart, Germany. *N*. The survival rate of rats was analyzed with Kaplan Meier method. The black line represents the survival curve of rAAV/IL12-treated rats and the dot black line represents the survival curve of PBS-treated rats.

Group 5 (n = 18):9 treated with AAV2/IL12 and 9 treated with PBS. Following these treatments, rats were implanted with tumor. Tumor growth was estimated from week 1 to week 3. (Figure 9)

Group 6 (n = 24):12 treated with AAV2/IL12 and 12 treated with PBS. Following these treatments, rats were implanted with tumor. The survival days were recorded for 8 weeks. (Figure 9)

### Statistical analysis

All data are shown as mean ± standard deviation. Student's *t*-test was used to compare two groups and one-way ANOVA with the Bonferroni post hoc correction was used to compare multiple groups. The survival time of the rat was analyzed by the Kaplan-Meier method and the Log-rank test.

## Results

### Effect of rAAV2/IL12 on IFN-γ and IL-12 expressions in the brain without tumor implantation

For evaluating effectiveness of rAAV2/IL12 injection in the brain in inducing an increased expression of IFN-γ and IL-12 in the brain, rats were treated with rAAV2/IL12 (n = 14) or treated with AAV2/GFP (n = 14) but not implanted with tumor. We evaluated time course of IFN-γ (Figure 1A) and IL-12 (Figure 1B) levels in both groups.

Figure 1A shows that the mean levels of IFN-γ in the right hemisphere of the rAAV2/IL12 treated rats with no tumor transplantation [rAAV/12 R (no) group] was increased from 33.5 ± 2.2 pg/mg prior to the injection to the maximum of 143.4 ± 15.5 pg/mg on the last day of week-6, and declined to 67.5 ± 21.7 pg/mg on the last day of week-8; in the left hemisphere, the mean concentrations of IFN-γ were increased from 31.2 ± 1.8 pg/mg prior to injection to the maximum of 88.2 ± 1.8 pg/mg on the last day of week-6, and declined to 40.0 ± 9.1 pg/mg on the last day of week-8. These findings indicated an ipsilateral increase in concentrations of IFN-γ (Figure 1A) in AAV2/IL12 R(no) group. On the other hand, the mean levels of IFN-γ in both left and right hemispheres of the rAAV2/GFP-treated rats with no tumor implantation [AAV2/GFP L(no) and AAV2/GFP R(no) groups] were all below 40 pg/mg, near the pre-treated level, indicating that rAAV2/GFP did not induce IFN-γ immunoresponse.

Figure 1B shows that the mean concentrations of IL-12 in the right hemisphere of the rAAV2/IL12 treated rats with no tumor implantation [AAV2/IL12 R(no) group] were increased from 3.4 ± 2.1 pg/mg prior to the injection to a maximum of 70.2 ± 12.3 pg/mg on the last day of week 4, but declined to 34.2 ± 19.3 pg/mg on the last day of week 8; the mean concentrations of IL-12 in the left hemisphere of the same rats [AAV2/IL12 L(no) group] were below 13 pg/mg at all time points. These findings indicated an ipsilateral induction of IL-12 expression in AAV2/IL12 R(no) group. The mean concentrations of IL-12 in either left or right hemisphere of rAAV2/GFP-treated rats with no tumor implantation [AAV2/GFP L(no) and AAV2/GFP R(no) groups] were all below 10 pg/mg throughout week 0 to week 8, indicating that rAAV2/GFP did not induce IL-12 immunoresponse.

Tumor implantation alone slightly increased the level of IFN-γ, presumably by activation of preexisting immune responses, and by infiltration into the contralateral hemisphere. The increase in IL-12 mediated by rAAV2/IL12 had a restricted distribution, since it only occurred in the hemisphere where the vector was injected.

### Comparison of rAAV2/IL12 effects on IL-12 and IFN-γ expressions in the brain with or without tumor implantation

We assessed the efficacy of our transfection system by measuring IL-12 in the right and left brain hemispheres of PBS-treated (n = 9) and rAAV2/IL12-treated (n = 9) rats on the last day of week 1, 2, and 3 after tumor implantation (Figure 2).

#### Effect on IL12

Figure 2A shows that mean concentrations of IL-12 in the right hemisphere of the rAAV2/IL12 treated rats (AAV2/IL12 RH group) were 53.9 ± 29.4 pg/mg, 83.8 ± 27.0 pg/mg, and 93.4 ± 18.1 pg/mg, while in the left hemisphere (AAV2/IL12 LH group), they were less than 13 pg/mg at all time points; on the other hand in both hemispheres of the PBS-treated rats (PBS RH and PBS LH groups), the mean concentrations of IL-12 were less than 16 pg/mg at all time points. In comparison among these four groups, the concentrations of IL-12 in the AAV2/IL12 RH group were significantly higher than those of the other three groups (*p < 0.001 *by ANOVA). Figure 2B compares the data from AAV2/IL12 RH group with AAV2/IL12 R(no) group, or compares those from AAV2/IL12 LH group with those from AAV/IL12 L(no) group, demonstrating that the concentrations of IL-12 were similar in either comparison. These findings indicated that our vector increased IL-12 expression and that this response was due to the vector that carries IL-12, and not a secondary response to tumor implantation.

#### Effect on IFN-γ

In the right hemisphere of the rAAV2/IL12 (AAV2/IL12 RH) group, the mean concentrations of IFN-γ were 87.7 ± 14.1 pg/mg, 118.7 ± 25.2 pg/mg, and 129.0 ± 21.6 pg/mg; in the left hemisphere (AAV2/IL12 LH), they were 68 to 86 pg/mg at all time points; while in both hemispheres of the PBS-treated (PBS LH and PBS RH) groups, they were 68 to 80 pg/mg at all time points (Figure 2C). In comparison among above four subgroups, the concentrations of IFN-γ in the AAV2/IL12 RH group were significantly higher (*p < 0.001 *by ANOVA) than those of the other three subgroups (Figure 2C). Figure 2D compares the data from AAV2/IL RH group with those from AAV2/IL R(no) group, or compares those from AAV2/IL LH group with those from AAV/IL12 L(no) group, demonstrating that the concentrations of IFN-γ were similar in either comparison (Figure 2D). These findings indicated that our vector ipsilaterally increased IFN-γ expression and that this response was due to the vector that carries IL-12, and not a secondary response to tumor implantation.

### Effect of rAAV2/IL12 on ED1 and TRAIL expression in the brain implanted with tumor

This study evaluated whether the treatment of rAAV2/IL12 can enhance an infiltration of the activated microglias by comparing expressions of ED1 and TRAIL in the brains treated with PBS and rAAV2/IL12 by Western blotting. We found that expressions of ED1 and TRAIL were ipsilaterally abundant in the rAAV2/IL12-treated rats (Figure 3B), but less in the PBS-treated rats (Figure 3A); these expressions were most prominent on the last day of Week 3 after tumor implantation. The ratio of ED1/actin and TRAIL/actin were detected of the last day of week 3 (n = 3, each group) (GS-800 Calibrated Densitometer, Biorad). The mean value of ED1/actin was 2.11 ± 0.27 of IL12-treated group and 0.95 ± 0.63 of PBS-treated group (*p < 0.05 *by *t*-test). The mean value of TRAIL/actin was 1.62 ± 0.63 of IL12-treated group and 0.67 ± 0.23 of PBS-treated group (*p < 0.001 *by *t*-test). These results indicate that activation of microglia and secretion of TRAIL are induced by the treatment of rAAV2/IL12.

Since expressions of ED1 and TRAIL in the rAAV2/IL12 and PBS treated rats were most prominent on the last day of Week 3 after tumor implantation, the following immunohistochemical studies on ED1, TRAIL, Ki67 and TUNEL staining were performed at this time point.

### Effects of rAAV2/IL12 treatment on immunohistochemistry for cell markers

For assessing immune responses induced by treatment of rAAV2/IL12, immunohistochemistry for cell markers including ED1, TRAIL, Ki67 and TUNEL were compared among brains treated with AAV2/IL12 (n = 4), AAV2/GFP (n = 2), PBS (n = 2), or treated with nothing (n = 1). Except for the rat treated with nothing, all other rats were transplanted with tumor on the last day of Week 2 after each treatment. Immunohistochemical staining was performed on the last day of week-3 following tumor implantation.

#### Effect on ED1 and TRAIL stainings

The infiltration of activated microglial cells was examined by immunohistochemical staining of ED1 and TRAIL, the markers of the activated. microglial cells. The infiltration of ED1- and TRAIL-positive cells was prominent, increasing from week 1 to week 3 in the rAAV2/IL12-treated rats, but it was sparse in the PBS-treated rats. Figure 4 shows that brain sections harvested from brains treated with rAAV2/IL12 (A, B, C, D) show small tumor size and prominent dark-brown ED1 positive cells infiltrating in four quadrants of the tumor; those with rAAV2/GFP (E, F) and PBS (G, H), show sparse dark brown ED1 positive cells infiltrating in four quadrants of the tumor; while the brain section treated with nothing (I) does not show dark-brown stained cells. These findings indicate that in the rAAV2/L12-treated rats, numerous activated microglial cells had clearly infiltrated the peripheral zone into the tumor; while in the PBS-treated and rAAV2/GFP-treated rats, only a few microglial cells infiltrated the tumor.

Figure 5 shows that brain sections harvested from rats treated with rAAV2/IL12 have prominent cells stained with dark brown TRAIL stain (A, B); those treated with PBS show sparse cells stained with dark brown TRAIL stain (C, D); that treated with nothing does not have cells show dark brown TRAIL stain (E). The pattern of infiltration of TRAIL positive cells into the tumor and its peripheral zone were essentially similar to that of the ED1 positive cells as in Figure 3. However, the intensity of the TRAIL stain was stronger in intercellular matrix. Although TRAIL stain was evident in the PBS group, the stain was much weaker (Figure 5C, D).

Furthermore, Figure 6 shows that in the rAAV2/IL12 rats, high power microscopy demonstrating that microglial cells were pleomorphic, ranged from hypertrophic and amoeboid to compound granular corpuscles, indicating transformation of microglia into active and mobile cells. However, most of the microglial cells in the PBS group were small and round or irregular in shape, indicating they were in the inactive form.

#### Effect on Ki67 stain

The activity of tumor proliferation was estimated by Ki67 stain. The cells with Ki67 stain were prominent in rAAV2/GFP treated tumors (Figure 7A, B) and only sparse in rAAV2/IL12 treated tumors (Figure 7C, D). In 10 high power fields of above brain sections of two rAAV/GFP-treated tumors, the mean Ki67 positive cells were 117 and 110, respectively; while in those of the two rAAV2/IL12-treated tumors, they were 26.3 and 16.8. These results indicate that cell proliferation in rAAV2/IL12 treated tumor was strongly inhibited.

#### Effect on TUNEL stain

The apoptosis of tumor cells was evaluated by TUNEL stain. The apoptotic cells were more rAAV2/IL12-treated tumors (Figure 8C, D) than the two rAAV2/GFP-treated tumors (Figure 8A, B). In 10 high power fields of above brain sections of two rAAV/GFP-treated tumors, the mean apoptotic cells were 16.2 and 33.4, respectively; while in those of the two rAAV2/IL12-treated tumors, they were 90.2 and 80.8. These results indicate that rAAV2/IL12 can induce more apoptosis of tumor cells.

### Effects of NO secretion of BV2 cells and stimulated by IL-12 in vitro

In the test of BV2 groups, the OD value was 0.046 ± 0.002 of blank, 0.045 ± 0.001 of 5 ng/ml IL-12 treated, 0.043 ± 0.002 of 10 ng/ml IL-12 treated, 0.044 ± 0.001 of 50 ng/ml IL-12 treated, and 0.254 ± 0.018 of LPS treated. There is no significant difference between IL-12 treated groups and blank, but significant difference between LPS treated and the others (*p < 0.001 *by ANOVA). This result indicates that LPS could induce BV2 cells to secrete NO, but IL-12 could not induce BV2 cells to secrete NO even in high dosage.

### Effect of rAAV2/IL12 treatment on tumor growth

Rats were treated with rAAV2/IL12 (n = 9) and with PBS (n = 9). Following these treatments, they were implanted with tumor (GBM). The tumor growth was not identifiable by gross view on the last day of week-1 for both groups; on the last day of week-2 (tumors had grown for 2 weeks), the rAAV2/IL12 group appeared to have small tumor (Figure 9A-C), but the PBS group had larger tumor growth (Figure 9D-F); on the last day of week-3, tumor growth was evident in both groups (Figure 9G-I and 9J-L). Tumor mass however was significantly greater and was able to invade into the contralateral hemisphere in the PBS group; occasionally, hemorrhage in the tumor was also noted (Figure 9J-L). Above data were summarized in Figure 7M. In the rAAV2/IL12 group, the mean tumor volume was 11.8 ± 6.0 mm^3 ^and 76.9 ± 9.7 mm^3 ^on the last day of week-2 and week-3, respectively; in the PBS group, mean tumor volume was 61.1 ± 13.7 mm^3 ^and 105.9 ± 9.6 mm^3 ^on the last day of week-2 and week-3, respectively. These differences were statistically significant, (p < 0.01 by *t*-test).

### Effect of rAAV2/IL12 treatment on survival rate of GBM-implanted rats

Rats were treated with AAV2/IL12 and with PBS (n = 12 each group). Following these treatments, rats were implanted with tumor. The survival days were recorded. The mean survival time was 27.2 ± 3.0 days for the rAAV2/IL12 group and 21.6 ± 1.3 days for the PBS group (Figure 9N). Kaplan-Meier analysis and the Log-rank test indicated that these differences were significant (*p < 0.00001*).

## Discussion

### Ipsilateral expressions of IFN-γ and IL12 by rAAV/IL12

The present findings demonstrate an ipsilateral increase in concentrations of IFN-γ (Figure 1A) and IL-12 (Figure 1B) in AAV2/IL12 R(no) group; other findings cannot demonstrate the induction of expression of IFN-γ (Figure 1A) and IL12 (Figure 1B) in AAV2/GFP L(no) and AAV2/GFP R(no) groups. Comparison among AAV2/IL12 RH, AAV2/IL12 LH, PBS RH, and PBS LH groups (Figure 2A and 2C), we demonstrate that the concentrations of IL-12 (Figure 2A) and IFN-γ (Figure 2C) in the AAV2/IL12 RH group were the highest and those of the other three groups were maintained at the baseline level. In addition, the concentrations of IL12 (Figure 2B) and IFN-γ (Figure 2D) were similar in both AAV2/IL RH group and AAV2/IL R(no) group. These findings together indicated that our vector ipsilaterally increased both IL-12 and IFN-γ expressions and that this response was due to the vector that carries IL-12, and not a secondary response to tumor implantation.

### Timing of expression of rAAV2-transfected genes

The timing of expression of vector-transferred genes in the brain is important in the treatment of different CNS diseases. The duration of transgene expression has not been established for therapy of different cancers, but it is expected to be shorter than that required for treatment of neurodegenerative diseases. Several previous papers have examined the expression of rAAV-transfected genes. Warren and colleagues [20] injected rAAV2-lacZ into the brains of BALB/c mice and found that β-gal expression increased for the first two months, decreased by month-4, and was not detectable in month-6 to month-15. Sharon and colleagues [21]. injected rAAV2 and rAAV2-based serotypes rAAV2/1, rAAV2/5, and rAAV2/8 encoding GFP into Sprague-Dawley rat brains. GFP expression was detected after four days in all four serotypes, reached a maximum in week-4, and was undetectable in month-9. Injecting rAAV2/IL-12 into rat brains without (Figure 1) or with (Figure 2) tumor implantation in the present experiment, we found IL-12 and IFN-γ were elevated from week-2 and peaked in week-6. The sustained high levels of IL-12 and IFN-γ demonstrate that our methodology may be suitable for cancer therapy.

### Expressions of ED1 and TRAIL by rAAV2/IL transfection

TRAIL is secreted from several kinds of immune cells, but secretion from microglia has only been recognized in recent years. In particular, Sermin et al. found that IFN-γ and lipopolysaccharide upregulated TRAIL secretion from a microglial cell line [22]. However, the intracerebral concentration of IL-12 needed to induce subsequent immune reactions mediated by the microglia is not yet known.

We demonstrated that expressions of ED1 and TRAIL were ipsilaterally abundant in the tumor tissue of rAAV2/IL12-treated rats (Figure 3A), but less in that of PBS-treated rats (Figure 3B), indicating that the rAAV2/IL12 transfection can enhance more infiltration of activated microglias that secrete ED1 and TRAIL in the transplanted tumor. These results were confirmed by other studies using immunohistochemical stainings of ED1-positive (Figures 4 and 6) and TRAIL-positive (Figures 5 and 6) cells. These studies demonstrated that the activated microglia stained with ED1 and TRAIL abundantly infiltrated in the transplanted tumor transfected with rAAV2/IL12.

### Effects of rAAV2/IL12 transfection on Ki67 and TUNNEL stains, tumor growth. And survival time

IL-12 and IFN-γ are potent cytokines to enhance microglial activity, and the activated microglias further express IL-12 and IFN-γ, resulting in a positive feedback-loop [23-25]. Actively growing tumors can prevent activated microglia from secretion of cytotoxic and apoptosis-associated cytokines [15,26]. However, our use of rAAV2/IL12 that increased the intracerebral concentrations of IL-12 and IFN-γ (Figures 1 and 2) and microglial infiltration (Figures 3, 4, 5 and 6) modulated the tumor environment, and restored the defensive function of the microglia. This notion is evident in the findings in Figures 7, 8 and 9.

The activity of tumor proliferation was estimated by Ki67 stain. The cells with Ki67 stain were prominent in rAAV2/GFP treated tumors (Figure 7A, B) and only sparse in rAAV2/IL12 treated tumors (Figure 7C, D), demonstrating strongly significant difference between rAAV2/IL12-treated and rAAV2/GFP-treated groups (p < 0.00001). On the other hand, the apoptosis of tumor cells was evaluated by TUNEL stain. The apoptotic cells were more prominent in the rAAV2/IL12-treated tumors (Figure 8C, D) than the rAAV2/GFP-treated tumors (Figure 8A, B). These results indicated that in rAAV2/IL treated tumor, cell proliferation was strongly inhibited, while cell apoptosis markedly enhanced. In consistent with these results, Figure 8 demonstrated that the tumor volume was significantly smaller, while survival time longer in the AAV2/IL12 transfected rats.

### Clinical implications: Localized adjuvant therapy

In currently clinical practice, malignant brain tumors are treated by surgical excision adjuvant with chemotherapy and/or radiotherapy [27]. The BCNU wafer has been successfully used as a local delivery agent for malignant brain tumors; this delivery is associated with less systemic toxicity, prolonged therapeutic window, and reduced time from surgical excision to drug administration [28,29]. The BCNU wafer sustains a high therapeutic level of bis-chloronitrosourea for only 2-3 weeks, not long enough to prevent tumor relapse several months later. In our rat model, however, intracerebral injection of rAAV2/IL-12 increased IL-12 and IFN-γ expressions for more than six weeks, suggesting that this method can provide longer immune surveillance. In addition, the resultant increase in activated microglia was associated with minimal toxicity in the area adjacent to the treated lesion, smaller tumor volume, and longer survival time. Our results suggest that rAAV2 vectors are feasible for somatic gene therapy of brain cancers.

## Conclusions

Intracranial injection of rAAV2/IL12 increases expression of IL-12 and IFN-γ in the brain implanted with tumor. The increased immunoactivity can inhibit tumor growth, and prolong survival time. Because rAAV2/IL12 requires few weeks of an incubation period and cannot totally eliminate cancer cells, this treatment should be considered as a possible adjuvant or concomitant therapy for brain cancers when accompanied by surgery and/or other treatment modalities.

## Competing interests

The authors declare that they have no competing interests.

## Authors' contributions

TLC carried out the biomolecular studies, animal model and drafted the manuscript. MJW participated in the design of the study and collection and analysis of the experimental data. CCS conceived the study and participated in the design of the study. All authors read and approved the final manuscript.
